# Treatment experiences and preferences of British South Asians living with recurrent depression

**DOI:** 10.1371/journal.pone.0348170

**Published:** 2026-04-29

**Authors:** Amirah Akhtar, Shabana Shafiq, Sahdia Parveen, Emmanuel Nwofe, Karen Windle

**Affiliations:** 1 Department of Applied Dementia Studies, University of Bradford, Bradford, United Kingdom; 2 Faculty of Health Studies, University of Bradford, Bradford, United Kingdom; 3 School of Nursing and Paramedic Science, Ulster University, Belfast, United Kingdom; RMIT University, AUSTRALIA

## Abstract

**Introduction:**

Depression is one of the leading causes of disability and is a highly recurrent condition. Recurrent depression has been associated with risk of suicide and greater morbidity. British South Asians are at greater risk of developing depression and face greater barriers to help-seeking and treatment uptake. To better support this population and reduce the risk of relapse it is important to explore treatment experiences and preferences.

**Aims:**

This study explored the treatment experiences and preferences of 12 British South Asians living with recurrent depression.

**Methods:**

We conducted semi-structured interviews and analysed the data using thematic analysis.

**Results:**

Pharmacological treatments were accompanied by a continuous lay evaluation process of the pros and cons. A preference for non-pharmacological interventions was found, particularly art-based, creative approaches. Interventions that were culturally adapted, with incorporation of religion were preferred. Barriers to recovery included inexperienced therapists and lack of support post-intervention to maintain durable effects of therapy.

**Discussion:**

Health professionals, such as general practitioners and therapists, play a key role in shared decision making, to ensure people with recurrent depression feel supported and culturally appropriate treatment is offered. Managing recurrent depression, including reducing the risk of relapse can improve quality of life, wellbeing and reduce the risk of co-morbid conditions.

## Introduction

Depression is the leading cause of disability worldwide [1] and is highly recurrent. Approximately 50% of people with depression experience a recurrence [2], often within the first six months of initial recovery. Prevalence rates for probable single lifetime episode of major depression has shown to be 6.4%, compared to probable recurrent major depression (moderate) at 12.2% and probable recurrent major depression (severe) at 7.2% [3]. A recent scoping review on lifetime prevalence of persistent and recurrent depression reported between 1.6–18% prevalence rate, largely impacting women [4]. Studies have shown health-related quality of life to be lower in people with recurrent depression in the remitted phase, compared to the general population [5]. This indicates that people with lived experience of recurrent depression require support in managing residual symptoms whilst in treatment, as well as ongoing support post-treatment.

Recurrent depression can have significant consequences on the individual with lived experience as well as wider public health consequences. A study found that self-reported suicidal symptoms persisted during the remitted phase of recurrent major depressive disorder [6]. This was a predictor for recurrence of depression. Alongside increased suicide risk, people with recurrent depression may also experience cognitive dysfunction, which includes challenges around memory, attention, and concentration [7–11]. This has shown to have a negative impact on psychosocial and occupational functioning [5,7]. Recurrent depression is also associated with economic burden, with mental health problems reportedly costing the UK economy £118 billion according to a recent study by the Mental Health Foundation and London School of Economics [12]. This indicates an economic need for effective treatments for depression, to reduce the risk of relapse and associated co-morbidities.

People from minority ethnic communities have shown to be at greater risk of depression. A study found prevalence rates to be higher amongst the British South Asian population compared to the White British population [13]. This was attributed to elevated disability risk in British South Asian communities. Despite higher prevalence rates, people of South Asian background are underrepresented in mental health services, such as NHS Talking Therapies (formerly known as IAPT), with only 6.5% receiving treatment for a common mental health disorder, compared to 9.7% of those from a White British background [14]. It is crucial to understand the reasons behind these inequalities in accessing treatment, by understanding past, current and future preferences of mental health support for minority ethnic communities. A study conducted in the UK found inequalities in the referral, assessment and treatment process of NHS Talking Therapies [15]. People from minority ethnic backgrounds were less likely to self-refer and were more likely to be referred by community services. People of minority ethnic background were also less likely to receive an assessment, and those who did were less likely to receive treatment [15]. Another study found practitioners to lack cultural competency, which can act as a barrier to treatment uptake and recovery rates [16]. Cultural differences in the construction, expression and experience of depression can sometimes lead to issues during identification and diagnosis [17,18]. Existing literature has long reported barriers such as stigma and mistrust of service providers to help-seeking and staying in treatment [19]. It is recommended by international guidelines that service users and clinicians work together to make decisions about treatment [20]. Receiving a preferred psychosocial intervention has been demonstrated to reduce dropout rates and improve therapeutic alliance [21]. Despite the evidence, service users continue to report dissatisfaction with the decision making process [22,23]. Developing an understanding of what service users may prefer and implementing this may be a first step in improving satisfaction rates and clinical outcomes.

One way to tackle these inequalities has been through culturally adapting depression interventions for British South Asian populations. Adaptions focused around cultural awareness of mental health issues, linguistic considerations, adapting content to reflect issues faced by the target population and culturally relevant materials, have shown to be effective in reducing symptoms of depression [24–26]. Currently, there is a lack of attention directed towards the issue of recurrent depression, particularly in minority ethnic populations who face greater challenges in the management and treatment of mental health issues. As discussed earlier, recurrent depression comes with significant impairment in both psychosocial and occupational functioning, impacting on quality of life and wellbeing. Furthermore, recurrent depression has been associated with co-morbid conditions such as diabetes and dementia [27,28].

To ensure people with recurrent depression feel supported, it is vital to understand their current experiences of treatment, in addition to exploring the treatment preferences of British South Asians with recurrent depression. This will help shape the future of services and interventions in a way that is culturally appropriate and effective for this population.

**Research questions:**

1. What is the experience of pharmacological and non-pharmacological interventions for British South Asians living with recurrent depression, and how can these inform culturally appropriate clinical practices?
2. What are the treatment preferences of British South Asians living with recurrent depression, and how can these inform the design of patient-centred, culturally sensitive mental health services?

## Materials and methods

A qualitative design was used to explore participants’ treatment experiences for recurrent depression via semi-structured interviews. This qualitative study is underpinned by an interpretivist epistemology and a constructivist theoretical perspective. It assumes that experiences of recurrent depression and preferences for treatment are not fixed or universal but are socially and culturally constructed. By focusing on British South Asian participants, the study recognises the role of cultural values, religion, family dynamics, migration histories, and interactions with healthcare systems in shaping how depression and treatment are understood. Ethics approval was granted by the research ethics panel at the University of Bradford, in the United Kingdom.

### Participant recruitment

Recruitment followed purposive sampling, convenience and snowball sampling of individuals identifying as British South Asian with recurrent depression. Whilst this approach was appropriate for capturing detailed, context-rich insights from the target population, it may introduce certain biases. Participants were recruited from community settings, such as community-based mental health support groups and mental health charities. The study was also advertised on social media. Whilst this decision was made to maximise recruitment, it may have resulted in a sample skewed towards individuals who are more engaged with mental health services. As such, the sample may not be fully representative of the broader British South Asian population with recurrent depression, especially those who face greater barriers to access or uptake of support. To ensure engagement with the target population and increase chances of recruitment, the first author visited community organisations in person to speak with service users about the study. Steps were taken to minimise sampling bias and enhance the diversity of perspectives captured. For example, efforts were made to include participants from different South Asian ethnic groups and religious groups. However, due to time constraints and geographical limitations, the area which we recruited from consisted of people from predominately Pakistani-Muslim backgrounds.

Whilst this sample specifically focused on the mid-to-older population, participants from each age bracket (40s, 50s, 60s) were recruited. This approach aimed to mitigate over-representation of any one subgroup and to capture a wider spectrum of experiences related to recurrent depression and treatment preferences. Interested individuals then received an information sheet and additional details of what taking part would involve. A total of 12 participants took part in this study (see Table 1 for participant characteristics). Informed consent was obtained from all participants prior to the interviews being conducted for their data to be used in research. Participants also completed a demographics form. Participants were not known to the lead researcher (AA) prior to study commencement.

**Table 1:** Participant characteristics.

Participant no	Gender	Age	Ethnicity	Religion	Education level	Occupation	Recurrent depression	Treatment	Language of interview
1	Male	50	Pakistani	Muslim	Post graduate diploma	Project manager	5 years	Non-pharmacological	English
2	Female	48	Indian	Sikh	BA (Hons)	Project manager	2 years	Non-pharmacological	English
3	Male	60	Pakistani	Muslim	No formal qualifications	Unemployed	25 years	Pharmacological and non-pharmacological	Pothwari
4	Female	56	Pakistani	Muslim	GCSE	Unemployed	5 years	Pharmacological and non-pharmacological	Punjabi
5	Female	58	Pakistani	Muslim	GCSE	Housewife	17 years	Pharmacological and non-pharmacological	English
6	Female	60	Pakistani	Muslim	No formal qualifications	Housewife	6 years	Pharmacological and non-pharmacological	Pothwari
7	Female	53	Pakistani	Muslim	GCSE	Daytime and Activities lead (Community centre)	3 years	Pharmacological and non-pharmacological	English
8	Female	46	Indian	Muslim	NVQ	Unemployed	31 years	Pharmacological and non-pharmacological	English
9	Male	48	Pakistani	Muslim	No formal qualifications	Unemployed	20 years	Pharmacological and non-pharmacological	English
10	Female	49	Pakistani	Muslim	MA	Unemployed	22 years	Pharmacological and non-pharmacological	English
11	Male	58	Pakistani	Muslim	GCSE equivalent	Unemployed	6 years	Pharmacological	Punjabi
12	Female	60	Pakistani	Muslim	GCSE equivalent	Housewife	31 years	Pharmacological and non-pharmacological	Punjabi

### Data collection

Interviews followed a topic guide, and questions were developed based on existing literature as well as discussions with the research team. The topic guide was shared with the PPI team for feedback (see section Patient and Public Involvement); however, it was not pilot tested. All interviews were conducted by the first author (AA) (female, PhD), an experienced qualitative researcher of South Asian (Pakistani-Muslim) background who is multi-lingual. Participants were made aware that AA is a research fellow on a dementia capacity-building fellowship. It was explained that AA is interested in dementia prevention, focusing on depression as a potentially modifiable risk factor. AA began this study with the appreciation that the lived experience of depression is culturally and socially situated. Whilst AA has previously conducted interviews with people of minority ethnic backgrounds and mood disorders, it was important to bracket prior assumptions. This included assumptions around the South Asian community, mental health stigma and help-seeking behaviours.

To mitigate potential interviewer bias, particularly given the shared cultural background between the interviewer and participants, AA engaged in ongoing reflexive practice. This included keeping a reflexive log to critically reflect on their positionality, assumptions, and how these may shape data collection and interpretation. For example, it was common for participants to refer to *‘you know what it’s like’* or *‘you know what I mean’* given the insider status of the researcher. In such cases, active listening and clarification strategies were used to allow participants to express their experiences in their own terms, while minimising imposition of the researcher’s perspectives. Regular meetings with co-authors provided an additional layer of critical scrutiny, enabling AA to interrogate whether emerging codes and themes were grounded in participants’ accounts or shaped prior expectations. Field notes were recorded during and after the interview and discussed with co-authors to ensure interviews captured information relevant to answering the research questions. The team included three further female (KW, SS, SP) and one male (EN) academics, all working within the field of healthy ageing. Two of the authors had outsider status (KW, EN), which balanced the perspective when findings and reflections were shared with the team.

Only the interviewer (AA) and the research participant were present during interviews. Seven interviews were conducted in English, three interviews were conducted in the official language of Punjabi, and two interviews were conducted in Pothwari, which is a dialect and not an official language. These were the languages participants felt most comfortable communicating in, as English was not their first language. Furthermore, people who do not speak English are often excluded from research and their voices are under-represented. A multi-lingual research team allowed their voices to be captured, contributing to the literature.

For participants who did not speak/read English, the consent form and information sheet were verbally translated. Seven interviews were conducted over the phone, three were conducted in-person at participants’ homes, and two were conducted online, via Microsoft Teams. All interviews were audio-recorded and transcribed verbatim. Participants took part in one interview, and there were no dropouts. Data was collected until saturation was reached. Saturation was conceptualised as meaning or theoretical saturation, in line with thematic analysis, referring to the point at which further interviews did not contribute new insights relevant to the research questions [29]. The analysis demonstrated that later interviews increasingly reiterated concepts identified earlier in the process. No new codes were added to the codebook during the latter interviews, and the coding framework had reached stability, with existing themes sufficiently captured across the sample. This indicated that additional data were unlikely to meaningfully alter or expand the thematic structure, suggesting that saturation had been achieved.

The translation process began prior to data collection; the topic guide was translated into Punjabi by a native speaker not involved in the study. Authors AA and SS translated the topic guide into Pothwari. The interviews conducted in English were outsourced to an external transcription company. The Punjabi interviews were also outsourced for transcription and translation into English. This was conducted by a native, in-country Punjabi speaker who was an expert in the topic of mental health and mood disorders. Once we received the transcripts, a native Punjabi speaker not involved in the study checked the accuracy of the translation. No changes were required.

The two interviews conducted in Pothwari were translated and transcribed by AA and SS, who are female, of Pakistani background and fluent in the Pothwari dialect. Given the linguistic and cultural specificity of the dialect, a hermeneutic approach to translation was adopted [29,30]. This process involved reviewing the original audio alongside the written English translation to ensure consistency, clarity, and interpretive alignment. It is important to note that subtle changes in words can occur even within the Pothwari dialect, dependent upon the region of origin, even if the regions are close in proximity. This may introduce interpretative inconsistency. As we were confident that AA and SS spoke the same version of the dialect, they collaboratively cross-checked each other’s translation. Translation was treated as an interpretive and dialogical process, prioritising conceptual equivalence over literal equivalence. This process facilitated a common understanding between AA and SS, reducing potential errors. Meaning was understood as contextually situated, shaped by cultural norms, regional variation, and interpersonal dynamics.

Transcripts were anonymised and checked for accuracy before the analysis, by AA. Transcripts were not returned to participants for comments, as over half of the interviews were conducted in a language other than English. All final transcripts were analysed in English, thus participant unable to read English would have been excluded during this process. The interviews varied from 30–60 minutes in length. All participants were debriefed at the end of the interview and received a £20 shopping voucher as a thank-you for their time. Participants were aware of payment via a shopping voucher prior to participation as stated in the participant information sheet. However, it was made clear that this is a thank you payment only and participants were not pressured to participate as a result of payment. Questions explored participants’ experience of current and past treatment for depression, including pharmacological and non-pharmacological interventions. This included what they thought worked well/didn’t work well in terms of reducing their depressive symptoms but also explored those treatments participants may prefer currently and/or in the future.

### Data analysis

Transcripts were uploaded into NVivo 12 Pro software for analysis and data was analysed using thematic analysis [30]. A deductive analytical approach was taken with the development of a preliminary coding framework using concepts from the interview questions. This included codes related to the type of treatment received, the experience of this, and treatment preferences, including barriers and facilitators to perceived engagement or effectiveness of treatment. To ensure reliability and rigour, two authors (AA and SS) coded the same four transcripts. Codes were then compared to discuss any discrepancies and to ensure consistent application of the coding framework. The remaining interviews were analysed by AA. Codes were then organised into themes reflecting the research questions and discussed with the research team. Participants did not provide feedback on the findings.

### Patient and public involvement

Individuals with experience of depression of British South Asian backgrounds were consulted during the development of this study. Four PPI members (2 male and 2 female) provided feedback on the proposed research aims and contributed to refining the topic guide to ensure that it was culturally sensitive, accessible, and relevant to the lived experiences of British South Asians. For example, they recommended including questions around how future treatment could be personalised to meet the needs of this population, with a particular focus around cultural adaptations. PPI members also recommended conducting interviews face-to-face where possible as interviews over the phone or online were perceived as less personable, acting as a potential barrier to participation. Offering further input on recruitment, they highlighted potential barriers to participation, including stigma around mental health and lack of familiarity with research processes, recommending an informal introductory session prior to data collection. As a result, AA conducted informal sessions with service users attending the wellbeing group at a community centre and peer support group at a mental health charity, to introduce the study, explain the research process, and answer questions. This was intended to build trust and reduce barriers to participation. A facilitator from the organisations were also present during the introductory session, as they were trusted figures this led to a good turnout of service users. The introductory session was received positively, and many service users voiced their interest in knowing more and potentially taking part.

## Results

Three overarching themes were developed that captured British South Asians experience of treatment for recurrent depression. The first theme explored experiences of pharmacological treatment, encompassed by a constant internal evaluation process made by participants, regarding the pros and cons. The second explored the positive and negative experiences of non-pharmacological treatments, moving on to challenges people face once therapy has ended and finally, a theme around treatment preferences. 12 participants took part, and all reported depressive symptoms from between 2–31 years (see Table 1.).

### Pharmacological intervention: Continuous lay evaluation process

Ten out of twelve participants in this study were taking medication to manage their depression. This theme captured how the use of anti-depressants is constantly evaluated by participants, which affected adherence. Participants were found to be involved in a continuous lay evaluation process, by which the pros and cons of anti-depressants were evaluated to determine their future choices.

*Direct experiential influence: perceived benefits and side effects*

Perceived benefits and side effects were consistently the most cited factors influencing adherence to medication. For some participants, medication was experienced as a useful treatment for managing symptoms of depression, contributing to improved wellbeing. Medication use was associated with reduced distress, supporting activities of daily living, increasing motivation and enabling a calmer state of mind. Participants reported the benefits of combined therapies (i.e., antidepressants plus exercise), noting that exercise alone, for example, was often insufficient to effectively manage depressive symptoms. These experiences underscore how perceived treatment benefits play a central role in participants’ ongoing evaluation of medication, reinforcing adherence when positive effects are experienced.

*I think that it kind of improves my motivation a little bit. Yeah, so I think in terms of day-to-day tasks (participant 10)*

*I am calmer, I can get on with my life. Before I used to be very anxious also, always worrying about the future. Now my mind feels clearer. (participant 8)*

*The doctor said “if you take it [anti-depressants], it’ll be for your benefit. If you don’t take it, you won’t get cured. You’ll get worse.” I do go out more since starting the medication, maybe that’s made a difference. (participant 3)*

*With the tablets, it was nice because your mind is fresh. But then for a month or two I didn’t take them because I said I wanted to do without them. So, I used to do a lot of walking. Then the walking stopped and I started to feel worse so I had to start the medication again. (participant 4)*

*Socially mediated influence: General Practitioners, family and friends*

Linking with side effects, the second most cited factor influencing adherence was on-going discussions with health professionals, namely General Practitioners (GP). When side effects were experienced, these were voiced to the GP with whom medication was reviewed, and in some cases an alternative dose of medication was prescribed to help counteract side effects. This led to a positive response and supported adherence to medication. However, dose adjustments were not always perceived as effective. Some participants reported repeated changes in dosage without experiencing improvement in their symptoms, which led to frustration and prompted reconsideration of the effectiveness of their treatment plan.

*They gave me a high dose, and it was so tiring that I used to feel tired and sleepy in the daytime, and then I stopped taking. Went back to the doctor, and I said this is what’s happening to me. The doctor said that could be a side effect. Then I said to her that I don’t want high dose, give me a lower dose, so I take lower dose now and that has helped. (participant 7)*

*I did mention it to the doctor that the medication wasn’t helping. And then all he did was sometimes increase it to 20 milligrams, and then sometimes he’d increase it to 60 milligrams and then decrease down to 40 milligrams. So all the doctor did was just adjust the dose high on law all the time. It didn’t benefit me. (participant 11)*

While responsive adjustments to medication could reinforce adherence, repeated dosage changes without perceived improvement prompted participants to question the effectiveness of their treatment.

However, seeking support from the GP was not always a positive and straight forward experience for all participants, which influenced how they evaluated and engaged with anti-depressant treatment. Due to factors such as fear of dependency, medication was not always a first choice for participants, but as discussed in the prior sub-themes, provided some benefit in supporting daily living. Interactions with GPs played an important role in shaping participants confidence in decisions made related to medication use. Some participants reported barriers to negotiating types of treatments being prescribed included feeling discriminated against and not being heard when suggesting alternatives to traditional medicines. Such experiences influenced how participants weighed the benefits of antidepressants against concerns about long-term reliance.

*I’ve had difficult experiences as well with GPs should we say in terms of having those conversations and again some of that has been about how I’ve looked and then some of that has been around disbelief and around some of the life experiences if you like. So, it’s been a mixture of things. So, it’s not really been that positive an experience for me to approach medical professionals and expect what I want to get if you know what I mean. I’m on [anti-depressants] at the moment but again I guess I don’t want to be dependent on it. I found that there’s a rush to give medication rather than looking at other therapies or other alternatives. So, there’s a bit of a lack of understanding of how some of those holistic methods can help. Medication can be great in the short term but I think in the long term I don’t want to become dependent on it. (participant 10)*

One participant in this study did not opt for medication to manage their depressive symptoms at the time this study was conducted. This was influenced by conversations with their GP, who suggested treatment at this stage may create a level of dependency. It can be assumed that symptoms may have been at the mild stage suggesting that symptoms can be managed by other (non-pharmacological) forms of treatment. Here, the risk of dependency on medication is communicated as outweighing any perceived benefits. The participant subsequently positioned medication avoidance as a prudent and clinically endorsed decision, stating, avoidance of medication in the future. This extract illustrates how authoritative medical discourse can significantly shape lay evaluation, reinforcing caution and legitimising non-use.

*It is something I would say I stayed away from because when I discussed it with my GP he was very clear that you’ve not had it up till now. I don’t want to start you on it now, because then there’s a level of dependency, I don’t want to create that at this stage. I would avoid drugs and medication as a form of treatment for depression. (participant 1)*

*It is something I would say I stayed away from because when I discussed it with my GP he was very clear that you’ve not had it up till now. I don’t want to start you on it now, because then there’s a level of dependency, I don’t want to create that at this stage. I would avoid drugs and medication as a form of treatment for depression. (participant 1)*

Overall, while perceived benefits and side effects remained central to decisions about adherence, interactions with GPs acted as an important mediating influence, shaping participants’ trust in treatment recommendations and their openness to continuing medication. Three participants described how the social environment influenced their ongoing evaluation of anti-depressant use. Being surrounded by family members and/or taking part in enjoyable activities acted as a protective factor in the social context. In such spaces, the perceived need for anti-depressants was minimised as participants felt better able to manage their symptoms, as improvements in mood were attributed to the supportive social environment rather than medication. However, participants emphasised that this influence was situational rather than decisive. When participants were removed from social spaces, they entered a lonely space, feeling isolated. Participants reported that this heightened their awareness of depressive symptoms and a renewed recognition of the need for medication. In these contexts, antidepressants were re-evaluated as necessary for maintaining stability, even when concerns around side effects remained.

*My adherence to the medication has been inconsistent; sometimes I take them, and other times I don’t. I did feel that taking the medication was necessary. However, during my time in Pakistan, where I was surrounded by family, I didn’t feel the need for antidepressants. The presence of my sisters, their children, and the overall family environment kept me happy and distracted from my depression. I was taken out and engaged in activities, which made a significant difference. But here, being alone often triggers my anxiety. I’m hesitant to take medication because of its potential health effects, but I recognize the necessity to use them to soothe my mind and brain. (participant 12)*

*When I’m around my children and grandchildren I feel happy. The house feels lively when they are here, I don’t think about my problems. Sometimes in them moments I think I am fine, I don’t need medication. But then they go home, for most of the week I am alone, it can be difficult. Then I think maybe it is best to stay on medicine. (participant 6)*

Another participant was introduced to counselling early on during their symptoms by a friend, which proved effective and thus negated the need for further (pharmacological) treatment.

My friend works at ******** told me about the counselling and how to get referred. I thought it would just be helpful talking to somebody. I think it’s been good for me. Now when there’s a counselling slot free I think someone else in need deserves it more than me. (participant 2)

Overall, whilst social environments influenced how participants interpreted their wellbeing, decisions about continued medication use were more strongly shaped by the perceived benefits of antidepressants and the management of side effects, with social support primarily altering how participants assessed their need for medication at particular moments.

*Interpretative framework: influence of religion*

Two participants rationalised the use of medication for depression management through religion. In some religions, such as Islam, there is a belief that there is a cure for every disease. Thus, anti-depressants in this case are constructed by participant and/or family members as a treatment available to ease symptoms of depression, which are created by humans, but ultimately provided by God. In this way, religion functioned as a facilitator of medication use, framing antidepressants as a valid and acceptable means of managing symptoms and supporting adherence.

*My family told me I should take medication because I wasn’t getting better. I tried to exercise to manage my mood but I couldn’t find the motivation. I thought why do I have to take medication? Why me? But my family said medication for depression is available, think of it as a cure from God. So I started taking. (participant 5)*

*I just thought, look, God gave medication. That’s what I thought, nothing else, because I follow the rules of the plan and what God said. (participant 9)*

### Experience of non-pharmacological intervention

The majority of participants in this study also had experience of taking part in non-pharmacological interventions. This was mainly peer support groups for depression via mental health charities or community centres which offered support to vulnerable individuals. This type of support was in a group format, and participants expressed their experiences of this.

#### Group support: the good and theerapist, religion and gend bad.

A positive of group support (mental health charity) included a sense of being understood as one is around people with similar experiences. The ability to connect with others led to feelings of not feeling alone in individual experience, which added to the benefits of attending group support sessions.

*When the group started, then people tell their feelings, you know, about families, bullying. I said, “Do you know what? This is my story”. I started liking it. I felt like, these are the people who understand. (participant 9)*

Another participant expressed the benefit of being encouraged to speak about what was causing them to feel depressed. The relaxed structure of the sessions meant there was space to speak generally about things to encourage connections and socialization, as well as specific conversations about depression. The group was described as encouraging and helpful, indicating that this was a safe space for service users to share their vulnerable experiences, to feel heard and understood.

*I used to go, it was really helpful. There were 2–3 women and a few other men so about five of us. We’d talk about anything for about 2 hours, talk about our depression, what’s going on for us, we would be encouraged to share our stories. (participant 3)*

However, group support was not perceived as suitable for everyone. For example, some participants described feeling more depressed as a result of being around other depressed individuals. The dullness of sessions could be related to the format, such as sessions tailored to talking only and not taking part in extra activities. This was reported as a barrier to attending sessions.

*I attended group sessions, but I found them to be quite dull. I preferred staying quiet and found myself feeling even more depressed after seeing others in worse conditions during a few visits to the centre. It worsened my own condition, so I chose not to return. (participant 12)*

Another participant described feeling judged in the group. The judgement was based on how the service user decided to dress. Wearing an Islamic dress, sometimes referred to as a Jabba or an abaya is usually a one piece that Muslim women wear over an outfit. The quote suggests that’s some women in the group associated this as not being dressed properly, i.e., indicating a sense of laziness in terms of dress sense. Feeling judged was reported as a barrier to attending, as this worsened their state when they were already struggling with their mental health.

*Then you’ll be judged “She wasn’t wearing clothes. Why is she wearing Jabba (Islamic dress)”? It’s my choice. The other lady goes, “Oh, they’re going to talk about it.” I say, “Let them talk about it. It doesn’t affect me.” I go, “It might affect you, not me. I want to wear what I want to wear, not what they like me wearing, you know?” So, you know, sometimes when you’re down, when you’re quite down in your moods, then you don’t want all people to be telling you off. Like, you’re not a child. You are older. (participant 5)*

#### Just talking doesn’t help.

Participants in this study had experience of Talking Therapies and for context purpose, participants attributed psycho-social causes to their depression, such as financial constraints, marital issues and perceived discrimination when navigating health and benefit systems. In such cases participants expressed a need for solution-oriented support that would, for example, improve their financial situation. In such cases, talking therapies were perceived as ‘talking for the sake of talking’, indicating a lack of perceived benefit from this type of support.

*For me it just feels like they [therapist] are just like repeating what you’re saying. It’s literally your words reflected back and it kind of can feel like you’re just talking for the sake of talking. Do you know what I mean? There’s no resolve. (participant 10)*

*I perceive it can be talking for the sake of talking. I prefer to focus on remedies or solutions rather than just have been talking therapy and sounding out. (participant 1)*

A desire to focus on the future through solution-orientated methods of support, without addressing past experiences was reported. Conversations regarding the past are ‘triggering’ and lead to a negative experience of talking therapy, as the individual is left feeling worse than they started. Participants were recruited to talking therapies through various avenues, via a social worker being one of them. It is possible that the expectations surrounding talking therapy included perceived and desired outcomes were not clearly discussed, contributing towards poorer uptake and outcomes.

*Counselling, I’ve been through that. But that was just talking, talking, talking, and that was annoying. Every time she used to ring, and it was the same thing, same thing. And I’d go, “That’s annoying me. You’re asking too many questions. And I didn’t want to talk that much. I didn’t want to go to counselling. I said, “You’re asking me. And then after, I’m thinking about what you’ve told me”. And so, I don’t want to. I want to calm down, I’m calming down, but you’re triggering it back up. And so, I stopped. The social worker put me there, you see. Do something so that we’re doing some work, not focusing on stupid ideas and what’s happened behind. (participant 4)*

However, other participants did experience benefit from talking therapies. As a symptom of depression is lack of motivation and interest in things you used to enjoy, talking this through with a therapist and finding small ways to make changes had a positive impact. In such cases, the therapist is supporting the service user to make changes within. Expectations of therapy to ‘fix’ external issues outside the remit of mental health services may lead to negative service user experiences and poorer outcomes for talking therapies.

*When they’re talking, they just talk, listen to me, and if I’ve got something, she suggests, ‘Okay, if you do this… If you feel that…’ because I don’t really find things, interesting. She said how to focus myself and make things interesting, and like when I am home, I don’t like doing anything. She said, ‘Why don’t you get dressed up? Your mood will go better.’ So, then I have a shower in the morning and I put new clothes on, and I feel better then. So, she’s helped me that way, or she’ll say, ‘Go out for a walk.’ I do like walking. I like to be with people. So, I feel happy. I went to town, and I met a few people that I knew, and on that day felt so good that it felt like my depression came out. (Participant 7)*

#### Inexperienced therapist/facilitators.

Another factor which acted as a barrier to staying in therapy included inexperienced therapists or group facilitators. This included not feeling supported by therapists. The quote below suggests that some therapy sessions were not well organised. Service users were sharing their vulnerable moments without enough time to talk about these issues in a supportive manner. The participant reported being cut off mid-sentence, which indicates a power imbalance as well as lack of human nature or empathy. The quote indicates these factors might be difficult for therapists to balance within time constraints of sessions.

*I was given a young therapist who seemed quite inexperienced. I was left feeling really vulnerable after one of the sessions. She did a really deep dive into my family and brought up a lot of stuff and then as I was talking she just literally cut me off and said I’m not sure if you’re aware but we’re at the end of our session now and that was it. That was the closure. Which just kind of didn’t leave me in a good place. (participant 10)*

Participants attending community centre support also reported similar themes around lack of empathy from facilitators. One of the symptoms of depression is lack of motivation to do things, thus participants reported difficulty around consistently attending sessions. A further barrier included the fear of being questioned if one did not attend. This implies that certain organisations did not feel emotionally safe for participants, as they felt misunderstood and needing to justify their absence. Whilst lack of motivation during depression might make it difficult for participants to take part in therapy, community support groups or interventions, the quote demonstrates that an empathetic approach towards this is more likely to support participants in attending.

*So obviously whoever is dealing with that group, “So what happened? Why didn’t you come?” You know, like, you know in the back of your mind, if you haven’t gone for two weeks, you’ll be questioned. The other centre I used to go to they would never ask you why you didn’t come. They understood that you know, a person can be going through things. The ladies they used to say they couldn’t be asked to get out of bed. Do you know what I mean? But it’s not accepted here. (participant 5)*

#### Remission of depression: struggles post-support.

Additional challenges people with lived experience in this study faced, included maintaining progress and recovery post-intervention. Participants described their depression as recurrent and lingering, which meant they never really felt that they recovered, instead it was always a work in progress. This might be one of the reasons why the lack of motivation, a symptom of depression, still impacted them post-intervention. This suggests that some individuals, particularly those with experience of recurrent depression, may require further support and check-in sessions post-intervention.

*I tried to keep going out and I did that for a week or two, but then after that I couldn’t do it anymore. I just didn’t feel like it. It was too much for me. It’s because he used to come to my home and encourage me to do things, to go out. I enjoy outdoors, but since he’s no longer with me, I just don’t feel motivated to do any of these things anymore. I managed to do things for myself, had a little bit of motivation after he left, but after that I just felt down again and I couldn’t do anything for myself. (participant 3)*

*I don’t feel that the benefits of the therapy as much as I did straight after the therapy, but that’s my own fault because I didn’t make time to do the exercises. Like there was the morning exercise where you remind yourself of all the positives in your life. So those kind of things to maintain it’s not always practical and easy. (participant 1)*

### Treatment preferences

As this sample had experience of both pharmacological and non-pharmacological treatments for recurrent depression, we were interested in knowing their preferred type of treatment and what this may look like when developing future interventions. Overall, a combination of pharmacological and non-pharmacological treatment was preferred.

*I feel that I need both the medication and the group. The medication can help with depression but the group I felt was also really helpful too. So, I think there should be both options available to help, a combination of both can help with depression (participant 10)*

Participants reported on the types and characteristics of non-pharmacological interventions they preferred.

#### Art and activities based therapy.

This included creative art-based activities, as well as physical activities. The rationale behind this, as demonstrated by the quotes, is that these types of activities keep people’s mind on the task/activity at hand, reducing the likelihood of ruminating thoughts during that time. The element of achieving or completing something (a fitness routine, a knitting project) was perceived as beneficial to overall wellbeing.

*I think sometimes having something that is more goal setting based is helpful. Sometimes the physical activity is kind of helpful. (participant 10)*

*I feel for me, because I sew clothes, that helps distract me from my condition. If I go outdoors that will also distract me. If this kind of activities can be made available that would help most women living with depression. (participant 6)*

*If we are like art, painting, decorating... to do something so I’ll be occupied doing something. So, for example, if we had a group session, where, in the initial sessions, people shared their stories and their backgrounds, would prefer something activity-based that helps boost your mood. (participant 4)*

Despite preference for and willingness to attend art-based/creative spaces of support for depression, the challenges around funding were noted by participants. This indicates a need for further funding in local, community-based centers/charities to ensure continuation of support that is demonstrating real-life benefit to service users.

*I said to her, “I used to knit”. She showed me how to knit. She brought knitting needles, wool and everything. You know what I mean? They used to do activities with us, you know what I mean? Which was really nice, you know. Meditation. That was very nice. We used to do that. We used to eat there if we felt like it. That was the good thing. And then they stopped the funding, you know, the centre. (participant 5)*

## Cultural adaptations

### Therapist factors

Some participants expressed that having a therapist of the same cultural and religious background to them would be beneficial. This is because a perceived core understanding of religious and cultural factors would be present and service users would not have to spend time and energy explaining concepts. The rationale behind this would be more constructive therapy sessions, whereby the behaviours and issues presented are not alien to the therapist.

*I think to have somebody of the same heritage that I wouldn’t have to explain those things. Like, for example, when my dad passed away that we had the funeral on the same day. You know, they’d have that understanding. There are certain things, just rituals and kind of culturally things that I feel like I wouldn’t have to over-explain or justify to somebody. I need somebody of the same faith sometimes, would be really helpful again just to understand those things so I don’t have to keep repeating them. (participant 10)*

Participants who did not speak English as a first language preferred a bilingual therapist and someone of the same cultural-religious background. A shared understanding of beliefs and values was integral for participants who did not speak English. Participants did not report any difficulties with a bilingual therapist being provided, however only four participants in this sample with experience of therapy, were non-English speaking.

*It needs to be someone who can speak Urdu or Punjabi so we can understand what is being said when we attend groups, because they can understand language and also our culture too. They have more understanding of us as people. (participant 9)*

However, others reported the drawbacks of a therapist from the same cultural or religious background, such as the risk of losing anonymity and confidentiality. In certain organisations it may be possible that such therapists are known to the service users and their wider family, who due to previous negative experiences express reservations and prefer someone from a different cultural or religious background. In such cases, anonymity and confidentiality (which should be standard) are higher in importance than shared cultural-religious understanding.

*It’s better to be speaking to a strange person, but the thing is they say to you that it’s confidential, but then some things do leak which shouldn’t. You know what I mean? Because a one-to-one who’s there? So, it’s either I’m the one who’s going to tell or you’re going to tell. (participant 4)*

### Religion

The majority of participants in this study were of Muslim background and expressed the potential benefits of incorporating Islamic teachings into therapy. Discussions around mental health from an Islamic perspective were associated with feelings of comfort and peace. Furthermore, some suggestions of activities were deemed culturally inappropriate or not what service users would prefer. As the quote suggests, going for a coffee to clear your mind might be the norm in some cultures or generations, but others may prefer turning to God or more spiritual avenues. Participants did not mention religion being incorporated into their previous therapy sessions. This is an increasingly popular and specialist area within mental health services, currently with limited training and workforce to offer such specialist services.

*Yes, I seek ease and help from Allah [God] and wish to move away from medication. Many women discuss similar issues [mental health] during Milad gatherings [mosque], which I find comforting and conducive to a peaceful atmosphere. (participant 12)*

*lf you’re Muslim, It’s not racist or something but, you know, with the Muslim people, they will understand it better than you. They’ll advise you that way. Do you understand? And obviously, with English people, they’ll say “Oh, you know, you should go out. Go for a cup of coffee, go for this”. Maybe you don’t want to do that. You know, that’s not in you. (participant 5)*

### Gender

In general, participants preferred a therapist of the same gender. A female participant expressed that the reasoning behind this was feeling more comfortable speaking to another woman.

*It doesn’t matter [cultural background], but I prefer a lady, because I feel comfortable talking to a woman than a guy. (participant 7)*

A male participant expressed a preference for a male therapist, as it was perceived that males take a logical approach to therapy. This might be referring to a directive approach to therapy, solution or goal orientated as opposed to psychotherapeutic approaches, which rely heavily on talking and getting to the depth of experiences.

*I feel comfortable with speaking to both male and female, but I resonate more towards males because I, without sounding sexist. But I see of men to be more logical in their approach, in their thinking. (participant 1)*

## Discussion

This study has demonstrated that British South Asians with recurrent depression require longer term, tailored, culturally appropriate support. The findings of this study indicate that the participants receiving pharmacological treatments for depression, namely anti-depressants, were constantly evaluating their need for medication. Resistance and inner conflict were present, which was ultimately overridden by the perceived benefits of medication to manage depressive symptoms. These findings are consistent with the wider literature on the experience of medication to manage various types of depression [31,32]. The decision or desire to stop or continue medication was personal and dependent upon individual circumstances. To ensure service users feel supported, it is important that GPs consider these personal circumstances when evaluating whether anti-depressants should be prescribed/continued. Shared decision making in health settings has been advised, particularly in recurrent depression [33]. The importance of understanding service user needs and involving them in the development of treatment plans in therapeutic settings was found. A key factor in treatment satisfaction lies within how the service user feels in each of these formats. Understanding the case history, particularly in recurrent depression, may help guide these decisions through a person-centred approach. This also applies to the type of therapy service users are seeking, such as goal-oriented or delving deeper into issues to understand the root cause of behaviours.

Participants acknowledged the need for a combination of pharmacological and non-pharmacological approaches to manage their depressive symptoms. In terms of non-pharmacological, some participants reported limited value in talk-based therapies, describing them as “just talking for the sake of talking” and not supportive, particularly when distress is framed in functional or relational terms rather than psychological terms. However, others found reminders and structured reflections during Talking Therapy to be useful. This mirrors findings in the literature that conventional Western talk therapies often feel misaligned with the expectations and needs of some South Asian clients [34].

Cultural norms around emotional expression particularly with people outside of the family, coupled with mental health stigma can discourage open dialogue in therapeutic settings. In South Asian communities, mental health distress is often experienced or communicated somatically or through social stressors [18]. Our study demonstrates greater emphasis on managing problems through practical means rather than private introspection (via counselling or talking therapies). Additionally, concerns were reported around confidentiality of sessions particularly when the therapist/counsellor is of a similar background to the service user. These concerns represent that of the wider literature on British South Asians and Talking Therapies [35]. It is also important to highlight that some participants did not feel ready for this type of therapy and were unwilling or did not see any benefit in talking about their issues. Some studies of cognitive behavioral therapy have found that higher expected engagement predicts symptomatic improvement [36]. Assessment of expectations surrounding a particular form of treatment could be a useful tool in predicting levels of engagement and potential symptomatic improvement.

By contrast, participants expressed an interest in art and creativity-based approaches to managing their depressive symptoms. These approaches were associated with attention and thoughts being re-directed to focus on something productive, thus improving mood. Activity-based approaches like art therapy allow emotional content to be externalised symbolically, reducing the need for direct verbalisation of distress and therefore lowering perceived social risk or stigma associated with disclosure. Creative practices may also resonate more strongly with cultural traditions of storytelling, symbolism, and embodied expression, offering familiar and less threatening avenues for engagement. Community arts initiatives have been used in South Asian settings to create culturally sensitive environments that facilitate connection and expression without the perceived intrusiveness of standard psychotherapy [37]. Currently, the evidence base for culturally adapted non-pharmacological therapies for depression, particularly for mid-to-older minority ethnic populations is lacking [26].

Participants were asked what factors were important to them if interventions were to be culturally adapted. A facilitator to attend therapy was having a therapist of the same ethnic and cultural background. This alignment was perceived to reduce the need to explain cultural or religious concepts during already time-constrained sessions, allowing more time for therapeutic work. Whilst more research is required to determine whether ethnic/cultural matching improves clinical outcomes, existing literature has reported benefits of ethnic/cultural matching in terms of engagement and satisfaction [38,39]. Language also emerged as a key factor, with non-English speakers expressing a strong preference for bilingual therapists. Prior literature has demonstrated that language acts as a barrier to help-seeking and remaining in treatment. Existing depression interventions, which have been delivered in the target language of the population have shown to be effective and acceptable [40–42]. This indicates that bilingual therapy leads to more effective treatment and outcomes. A 2023 report on ethnic health inequalities in Improving Access to Psychological Therapies stated that data on preferred language was not available but would be available in future reports to allow services to understand the impact of language, bilingual therapists, as well as use of interpreters [43]. It must be noted, however, that these preferences may raise several ethical and systemic challenges. In small or close-knit communities, ethnically or linguistically matched therapists may be known to the client, resulting in concerns about confidentiality and trust [44]. Ethical codes caution against dual relationships and stress the need to protect client privacy. These issues must be at the forefront and carefully negotiated during the development of treatment plans.

Overall, participants in this study preferred being matched by gender. This is in line with previous research, which found gender matching to be a predictor of satisfaction with therapy [45]. Furthermore, research has also found greater chances of uptake of therapy when matched by gender [46]. Another study found minority ethnic populations to prefer gender matched therapists compared to their White counterparts [47]. This indicates that this component is important when developing a treatment plan for service users. The sample of this study comprised of an Islamic religious background, with a preference to incorporate teachings of Islam into therapy and/or delivering mental health support at religious settings such as mosques. Religion can be a sensitive topic as each person varies in their religiosity and spirituality. However, studies have shown the incorporation of religion into therapeutic settings to have benefits in reducing depression [24,48]. As found in this study, it is important that any examples or advice given are culturally appropriate and fit within the service user’s current lifestyle norms. For example, when understanding a service user’s background and day to day life, it might be more appropriate to tailor advice within a religious or spiritual framework. Whilst preferences of service users are important to consider as they may influence clinical outcomes, they also come with systemic and ethical challenges. All therapists regardless of their background should be appropriately trained in offering culturally competent care. As our findings suggest, each service user is different and thus, we run the risk of stereotyping or generalising a person’s needs based on their culture, religious background or gender, rather than addressing personal therapeutic fit [49].

One of the main issues reported in this study included maintaining recovery, particularly after therapy. A barrier to this was finding the motivation and time to implement exercises and skills learnt in therapy. Maintenance anti-depressants are a common preventive measure; however, in the current study adherence was found to vary, which may worsen depression symptoms. Education on the consistent use of anti-depressants is recommended to ensure people living with recurrent depression are managing their symptoms appropriately and safely. In addition to this, participants could benefit from on-going therapeutic/non-pharmacological support. A study found preventive cognitive therapy in addition to anti-depressants offered greater protective effects than anti-depressants alone [50]. The results of this study also suggest that top up sessions and/or preventive cognitive therapy should be offered to service users who are approaching the end of therapy. It is also recommended that service users are signposted or referred to existing community-based initiatives to support wellbeing. These services are often culturally tailored, offering a range of support from creativity based to social activities and peer support.

### Implications for practice

This study suggests that decisions regarding adherence to pharmacological treatments for depression are shaped by the individuals’ on-going personal evaluations, including perceived benefits, side effects and influence of spirituality, family members, friends and GPs. It is important for clinicians to recognise the influence of cultural and social context on treatment decisions. Collaborative conversations that address concerns about medication and incorporate patients lived experiences may improve trust, adherence, and treatment engagement. It is also vital for practitioners to be open to alternative forms of treatment, and to take an inclusive approach to treatment planning to ensure that the service user feels a sense of autonomy and empowerment in their treatment decisions.

When referring British South Asians to talking therapies or counselling-based support, it is recommended that expectations and readiness to receive such support is assessed. Whilst such approaches can be beneficial, this is dependent upon what the service user expects to gain from sessions, which can be influenced by their cultural background, norms and values. Clinicians may therefore consider incorporating more structured, practical, or solution-focused elements within therapeutic approaches. A person-centred approach is recommended, without preconceived ideas of what British South Asians prefer. For example, spirituality is deeply personal and should therefore be discussed whether this is something a service user wants in their treatment plan.

Mental health services may benefit from integrating follow-up support, relapse prevention strategies, or community-based resources that provide continued guidance beyond the end of formal treatment. Further research is required to understand what this may look like and whether it is feasible.

### Strengths and limitations

A strength of this study is that to our knowledge, it is one of the first studies to explore recurrent depression in British South Asians. This has deepened our understanding of which treatments might be more receptive to this population to ensure better outcomes, including support strategies we can apply to reduce the risk of relapse. A limitation of this study is that the sample lacked diversity in terms of ethnic and religious background Due to time constraints, recruitment was limited to an area with a higher proportion of Pakistani-Muslims, resulting in a recruitment bias. Thus, the experiences reported may not be representative of all British South Asians, affected the generalisabiltiy of findings.

A further limitation relates to potential interviewer and interpretive bias. As a South Asian researcher interviewing South Asian participants, shared cultural background may have shaped both the interaction and interpretation of narratives. While this positionality may have facilitated rapport and openness during interviews, it may also have introduced assumptions or shared understandings that influenced the analytic process. While reflexivity and positionality were considered throughout the research process, the possibility of researcher influence on data generation and interpretation cannot be entirely excluded.

It is also important to recognise that the findings are based on participants’ self-reported experiences, which can be subject to several biases. These include recall bias and social desirability bias, where participants may unintentionally misremember or respond in ways they perceive to be more acceptable. For example, it is possible that participants expressed a preference for religion or cultural matching as they were being interviewed by someone of that religion/background, possibly displaying social desirability [51,52]. Future studies should aim to include a wider range of ethnic, linguistic, and religious identities to better reflect the heterogeneity within this population.

## Conclusion

Recurrent depression is a public health concern, particularly in minority ethnic communities who are at greater risk and face additional barriers to accessing support. To reduce health inequalities and improve services, we explored the treatment experiences and preferences of British South Asians. Pharmacological treatments were constructed as being necessary but not without a continuous lay evaluation process. A greater preference for non-pharmacological and culturally adapted treatments were found for recurrent depression. As this population is at greater risk of relapse, it is vital that post-therapy support is considered and implemented. Health professionals including general practitioners and therapists play a pivotal role in shared decision making, vital for individuals with recurrent depression who may have different support needs compared to those with single episode depression. Effective management of recurrent depression through a multi-disciplinary approach has the potential to improve quality of life outcomes and reduce the risk of co-morbidities.

## Supporting information

S1 File COREQ checklist PLOS ONE 2026. (DOCX)
